# Cellular Contact Guidance on Liquid Crystalline Networks with Anisotropic Roughness

**DOI:** 10.1021/acsami.2c22892

**Published:** 2023-02-15

**Authors:** Marta Rojas-Rodríguez, Tania Fiaschi, Michele Mannelli, Leonardo Mortati, Federica Celegato, Diederik S. Wiersma, Camilla Parmeggiani, Daniele Martella

**Affiliations:** † European Laboratory for Non-linear Spectroscopy, via Nello Carrara 1, 50019 Sesto Fiorentino, Italy; ‡ Department of Biomedical, Experimental, and Clinical Sciences “Mario Serio”, University of Florence, viale Morgagni 50, 50143 Florence, Italy; § Istituto Nazionale di Ricerca Metrologica (INRiM), strada delle Cacce 91, 10135 Turin, Italy; ∥ Department of Physics and Astronomy, University of Florence, via Sansone 1, 50019 Sesto Fiorentino, Italy; ⊥ Department of Chemistry “Ugo Schiff”, University of Florence, via della Lastruccia 3−13, 50019 Sesto Fiorentino, Italy

**Keywords:** liquid crystalline network, anisotropic roughness, biomaterials, myotube differentiation, cell alignment

## Abstract

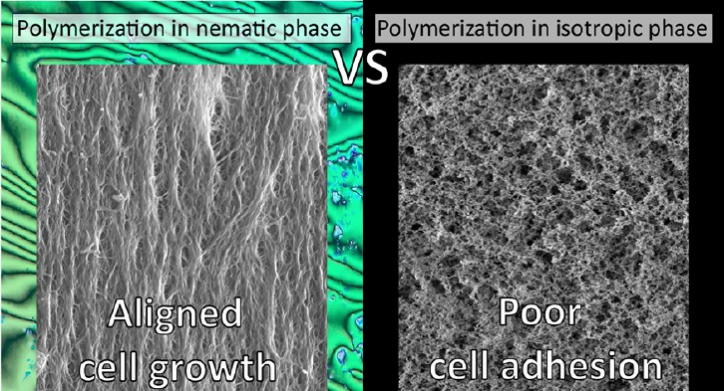

Cell contact guidance is widely employed to manipulate cell alignment and differentiation *in vitro*. The use of nano- or micro-patterned substrates allows efficient control of cell organization, thus opening up to biological models that cannot be reproduced spontaneously on standard culture dishes. In this paper, we explore the concept of cell contact guidance by Liquid Crystalline Networks (LCNs) presenting different surface topographies obtained by self-assembly of the monomeric mixture. The materials are prepared by photopolymerization of a low amount of diacrylate monomer dissolved in a liquid crystalline solvent, not participating in the reaction. The alignment of the liquid crystals, obtained before polymerization, determines the scaffold morphology, characterized by a nanometric structure. Such materials are able to drive the organization of different cell lines, e.g., fibroblasts and myoblasts, allowing for the alignment of single cells or high-density cell cultures. These results demonstrate the capabilities of rough surfaces prepared from the spontaneous assembly of liquid crystals to control biological models without the need of lithographic patterning or complex fabrication procedures. Interestingly, during myoblast differentiation, also myotube structuring in linear arrays is observed along the LCN fiber orientation. The implementation of this technology will open up to the formation of muscular tissue with well-aligned fibers *in vitro* mimicking the structure of native tissues.

## 1 Introduction

The interaction between cells and synthetic materials represents one of the main aspects to be faced in reproducing biological models *in vitro*. Not only do scaffolds represent surfaces for cell anchoring, but they are also able to direct the biological response, addressing cell organization and promoting specific morphogenetic pathways.^1^ In this regard, the scaffold and the cells establish a strong interplay where chemical and physical stimuli are sensed by the cells from the surrounding material and used in the formation of a specific tissue.^2^ Studying the cell–scaffold interactions (for both 2D and 3D models) is very interesting because cell cultures *in vitro* play a central role for biological and medical research, for both physiological and pharmacological assays.^3^

In the development of a scaffold, it is crucial to reproduce the complex extracellular matrix (ECM) of the native tissues—having anisotropic structures capable of driving specific cell organization, which, in turn, determines the tissue function.^4^ A simple example is the hierarchical structure of skeletal muscles, where myofibers are uniaxially aligned to give efficient contraction. Between the natural cell organization, the most common are the uniaxial alignment (e.g., in peripheral ligament and tendons or skeletal muscles), the platelike multilayered alignment (e.g., in myocardium or articular cartilage), and the tubular-shaped multilayered alignment (e.g., in the annulus of intervertebral disks or some blood vessels).^5^ The first one is for sure the easiest to reproduce *in vitro*,^6^ for example, by cyclically stretched,^7,8^ electrically stimulated,^9^ or specifically patterned^10^ scaffolds. However, the use of the above-mentioned stimulated scaffolds presents strong limits about the technology scalability because of the complex apparatuses and protocols needed. On the other hand, the use of patterned substrates is easier for the end users (biological and medical laboratories), and it has benefited from the great advances in nano- and micro-fabrication techniques in the last 30 years.^11^ With these substrates, the orientations of the cells and stress fibers are directly manipulated, designing the geometrical surface pattern with a phenomenon generally called cell contact guidance.^12^ Trying to mimic the length scale and structure of *in vivo* topographies, several studies have been concentrated on the minimal feature size for influencing cell behavior^13^ and identify whether a nano- or a microstructuration is more efficient for cell manipulation.^14^ Indeed, the scaffold surface (and the ECM *in vivo*) can influence the cell’s fate both at the nanometer length scale (mainly affecting the subcellular behaviors such as the organization of molecular receptors) and at the micrometer one (mainly affecting the cell morphology, cell migration, and tissue organization).^15,16^

However, a general guideline for determining the best scaffold morphologies is difficult to highlight, and different reports suggest that the better choice between nano- or micro-structuring mainly depends on the cell type.^17^

Cell contact guidance is a method to manipulate the cell alignment also used in physiological studies and diagnostic assays. A nice example reported the very different orientations and structures of human-induced pluripotent stem cells derived from patients affected by Duchenne Muscular Dystrophy, when seeded on Matrigel substrates patterned with nanogrooves, if compared with those from healthy patients, thus allowing for discrimination between normal or diseased cells.^18^

Still this field of research would incredibly benefit from simpler fabrication setups, hence allowing the formation of large area patterns, for example, by self-assembly methods. A powerful method for the spontaneous anisotropic pattern formation is based on polymer-stabilized liquid crystals (LCs) where mesogenic monomers in low amounts are mixed with nonreactive LCs and then polymerized.^19^ The network structures were found to be related to a transfer of the orientational order of the mesophase on the growing polymer chains, and, very interestingly, this effect has been previously observed also in the case of nonmesogenic monomers, e.g., to obtain anisotropic hydrogels^20^ or for templating of inorganic materials.^21^ Playing on the nonbounded LC content in the initial mixture, control of the pore structure has been described to prepare ultrafiltration membranes.^22^

The use of self-assembled patterns made by LCs as scaffolds have already been reported for fibroblasts on a linear groove array prepared by a smectic phase, both in polymers^23^ or low-molecular-weight LCs^24^ and for neural tumor cells.^25^

Moreover, the use of liquid-crystalline networks (LCNs) as cell instructive materials to induce cell alignment has been reported also on flat films or 3D scaffolds.^26,27^ LCNs are materials well-known for their properties as artificial muscles^28,29^ and exploited in several applications from robotics to photonics.^30,31^ Their use as cell scaffolds started only a few years ago,^32^ demonstrating their ability to induce human fibroblast and murine C2C12 myoblast alignment along the LC director^33,34^ and to promote the formation of myotubes with improved electrophysiological properties with respect to those obtained on the classic Petri dishes (commonly used in all biological laboratories).^35^ However, these systems call for optimization (for example, the cell alignment is limited to very high confluence) and need to be exploited for other cell lines.

Trying to move toward simpler and general methods for cell alignment and differentiation, we explored self-assembled LCN coatings as systems for cell contact guidance. We report here the fabrication of rough surfaces made by LCNs focusing on how simple fabrication parameters, such as the polymerization temperature and network composition, can be adapted to modify the cell adhesion and to improve the alignment process on the scaffolds.

## 2 Results and Discussion

**Figure 1:**
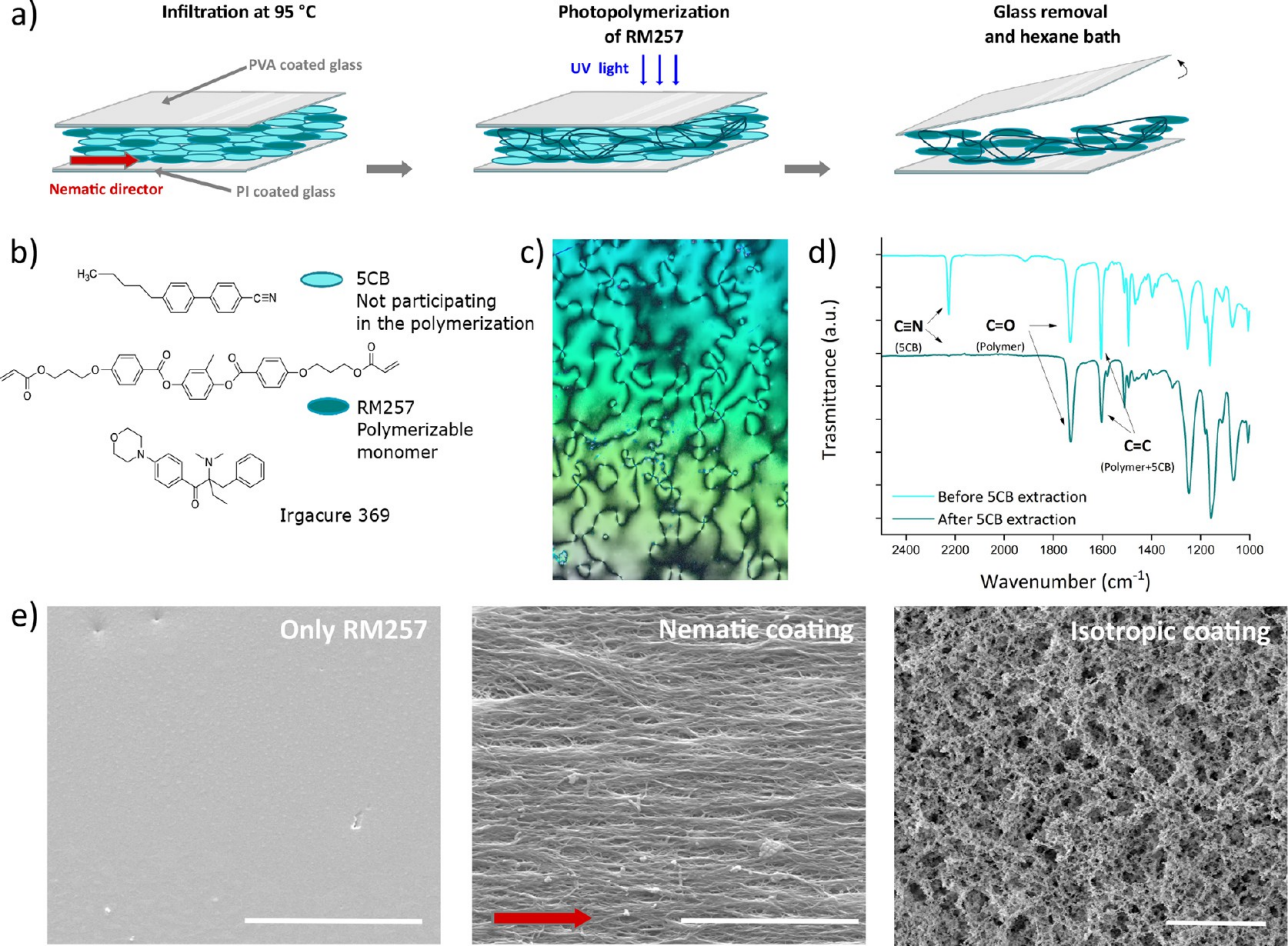
Preparation of LCN coatings with anisotropic roughness. (a) Description of the steps for material preparation. (b) Structures of monomeric compounds. (c) POM image of the monomeric mixture at room temperature before the aligning process. A typical nematic Schlieren texture (with the simultaneous presence of 2-arm and 4-arm defects) is shown. (d) ATR-IR spectra of the final material before (light-blue spectrum) and after (green spectrum) 5CB extraction. (e) SEM images of a flat LCN (polymerized in the nematic phase and only containing RM257), a LCN coating prepared in the nematic phase (the red arrow indicates the nematic director), and a coating prepared by the same mixture polymerized in the isotropic phase. Scale bars: 10 μm.

The self-assembled scaffolds have been prepared by photopolymerization following the steps depicted in Figure 1a. The monomeric mixture (Figure 1b) was composed of 4-pentyl-4-biphenylcarbonitrile (5CB), a well-known room temperature LC, and the diacrylate mesogen RM257 [5% (mol/mol) with respect to 5CB]. Irgacure 369 was added to control the polymerization by UV light. The mixture presented a nematic phase with a clearing temperature of 40 °C according to polarized optical microscopy (POM) observation (Figure 1c, reporting a nematic Schlieren texture). It has been infiltrated in a LC cell composed of two glasses treated to induce a homogeneous planar alignment of LCs. In particular, we used a poly(vinyl alcohol) (PVA) coating on one glass and polyimide (PI) on the other, both rubbed unidirectionally with a velvet cloth and separated by 50 μm spacers. PI was used to obtain enhanced adhesion on one glass where the final coating remained after the washing steps. After mixture infiltration, a homogeneous planar alignment has been obtained by cooling the cell at room temperature, and the sample has been polymerized by UV light. During irradiation, the radicals formed by the photoinitiator allow for the free-radical polymerization of RM257 (bearing the polymerizable acrylate group), thus forming a LC gel with 5CB (acting as a liquid-crystalline solvent). To obtain a uniform coating, it was essential to remove the top glass (without damaging, scratching, the soft LC gel). This was possible, and spontaneously obtained, by dissolving the PVA layer over 2 days in a water bath. The LC gel remained attached to the other glass (the one coated with PI). Afterward, the material was washed in hexane to remove 5CB, dried, and treated with a plasma cleaner to remove possible impurities. The thickness of the final coating was around 1 μm, thus showing a collapse of the polymeric architecture after 5CB removal (Figure S1).

Interestingly, the polymeric network retained the homogeneous planar LC alignment as observed by POM (Figure S2) and a milky aspect due to the presence of air within the fibrillar structure. The same procedure has been repeated by polymerizing the cells in the isotropic phase (90 °C), and, as expected, the material did not present any birefringence (and therefore alignment) by POM (Figure S2).

A critical aspect to test such materials as the cell scaffold is related to the correct removal of 5CB, which could lead to toxicity problems at high concentrations.^36^ Using attenuated-total-reflectance infrared (ATR-IR) spectroscopy, complete removal of this compound has been monitored, as shown in Figure 1d. After polymerization, the spectrum (light-blue line) showed typical vibration bands related to functional groups of both the polymer and LC solvent. In particular, 5CB presents a strong band at 2225 cm^–1^ attributed to the stretching of the −CN triple bond, while the polymerized RM257 shows a characteristic −C=O stretching at 1725 cm^–1^. The stretching of the aromatic C=C bond at 1695 cm^–1^ derives from both compounds. After a hexane wash, the spectrum (green line) did not show any more −CN stretching, while the intensity of the C=C stretching was lower (when normalized with the intensity of C=O) than that in the initial material, thus indicating successful removal of the unpolymerized LCs.

The morphology of coatings polymerized in the nematic or isotropic phase has been studied by scanning electron microscopy (SEM) and compared with that of flat LCN previously tested as cell scaffolds.^35^ The latter polymers beeing prepared with the same protocol but without 5CB in the initial mixture.^37^ Exemplificative SEM images are reported in Figure 1e, and more pictures at higher magnification are shown in Figure S3. The LCN prepared without 5CB presents a bulk structure with a flat surface and without pores. In contrast, the other materials, prepared in the presence of a high amount of 5CB, presented rough surfaces with two distinct textures depending on the polymerization temperature.

The material prepared in the nematic phase was composed of a network of smooth strands, with fibers parallel to the glass surface and mostly aligned in the direction of the LC director. The medium fiber diameter was between 80 and 180 nm (more details on the fiber dimension statistic are shown in Figure S3). To confirm the strand orientation dependence from the LC director, the same material has also been polymerized in the nematic phase using a cell coated with PVA without rubbing. Also in this case, anisotropic fibers parallel to the coating surface were observed, but their disposition was not unidirectional anymore (Figure S4), while it followed a spontaneous direction of liquid crystals inside the polymerization cell (polydomain samples). This experiment confirmed the importance of the mesogen alignment in the monomer mixture to address specific coating organization.

The situation was very different when the polymerization occurred under isotropic conditions. In this case, the templating effect of 5CB was not present, and the surface was no longer composed of fibers while a rice grainlike texture was formed (with a medium grain size of 76 ± 3 nm). The coating structure was also very different from the bulk film (obtained by the polymerization of RM257 alone) because the high percentage of nonreactive LCs induced a porous structure.

It should be noted that, even if the porosity was present through the entire material thickness (Figure S3), the pore size was much smaller than the cell dimensions and then the scaffolds have to be considered for 2D cell cultures (where cells grow, forming a single layer on top of the material). In the rest of this paper, we refer to nematic and isotropic coatings, respectively, for the two samples described above.

More insight on the scaffold morphology has been obtained by atomic force microscopy (AFM), and images of the different coatings are reported in Figure S5. From this analysis, the material roughness was measured as the absolute-mean-average roughness (*S*_a_) and root-mean-square roughness (*S*_rms_), resulting, for nematic coatings, about 75 and 95 nm, respectively, and, for isotropic coatings, about 96 and 124 nm, respectively. It has to be noted that this roughness definition does not take into account the anisotropy of the surfaces, which, on the other hand, is clearly shown by Figure S5 for the nematic coating.

The different scaffold morphologies should lead to differences in their mechanical properties, cell adhesion, and differentiation ability, as validated in this study.

**Figure 2:**
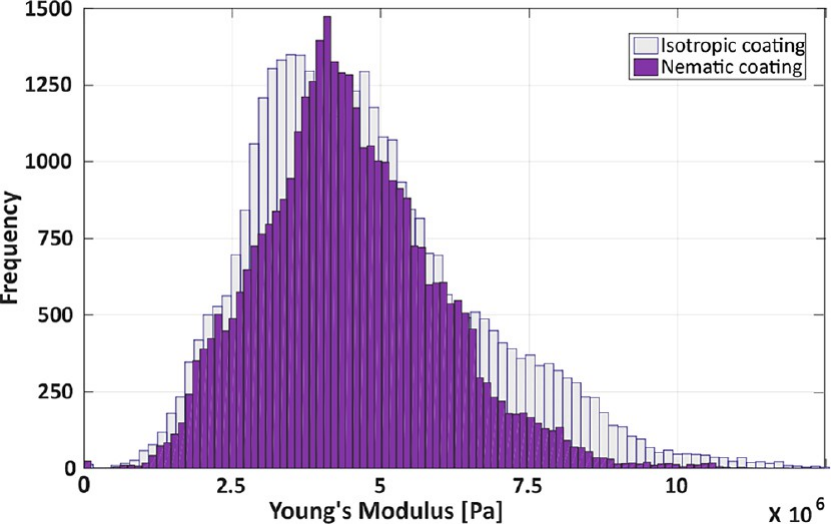
Mechanical properties of the LCN coatings measured by AFM. The histograms show the frequency of the Young’s modulus measurements in both isotropic (gray) and nematic (purple) LCN coatings.

Starting from the scaffold mechanical properties, the surface stiffness has been studied by AFM (Figure 2). For both the isotropic and nematic coatings, the measured Young’s modulus has a similar distribution, with more probable values located at around 3–4 MPa, even if the isotropic coating has a slightly lower Young’s modulus than the nematic one.

The biological tests have been focused on demonstrating how the LCN coatings were able to modulate the adhesion and the organization of different cell lines such as the human dermal fibroblast (HDF) and C2C12 murine myoblast. These two cell types were carefully chosen to test the cell contact guidance effect on cells coming from different organisms (human and murine) and characteristics of different tissues (connective and skeletal muscle types). Also, the type of cell culture is different, being a HDF primary cell culture, whereas C2C12 is an immortalized myoblast cell line. A known number of cells were seeded on the LCN coatings and on the Petri dishes, used as a control, and allowed to grow a few days (2–3 days) until the cells reached different confluency percentages (approximately from 60 to 100%).

The initial adhesion was similar in all the materials analyzed. Later on, good viability and proliferation was observed only for the nematic coating and Petri dish, showing very similar cell densities (reported in Table S1 after 3 days of culture) for both cell lines (thus indicating a similar rate of cell growth). In contrast, on the isotropic coating, a very low cell density was observed. This first observation demonstrates how the material structure is a further parameter to be considered besides the material composition in determining the cell fate.

**Figure 3:**
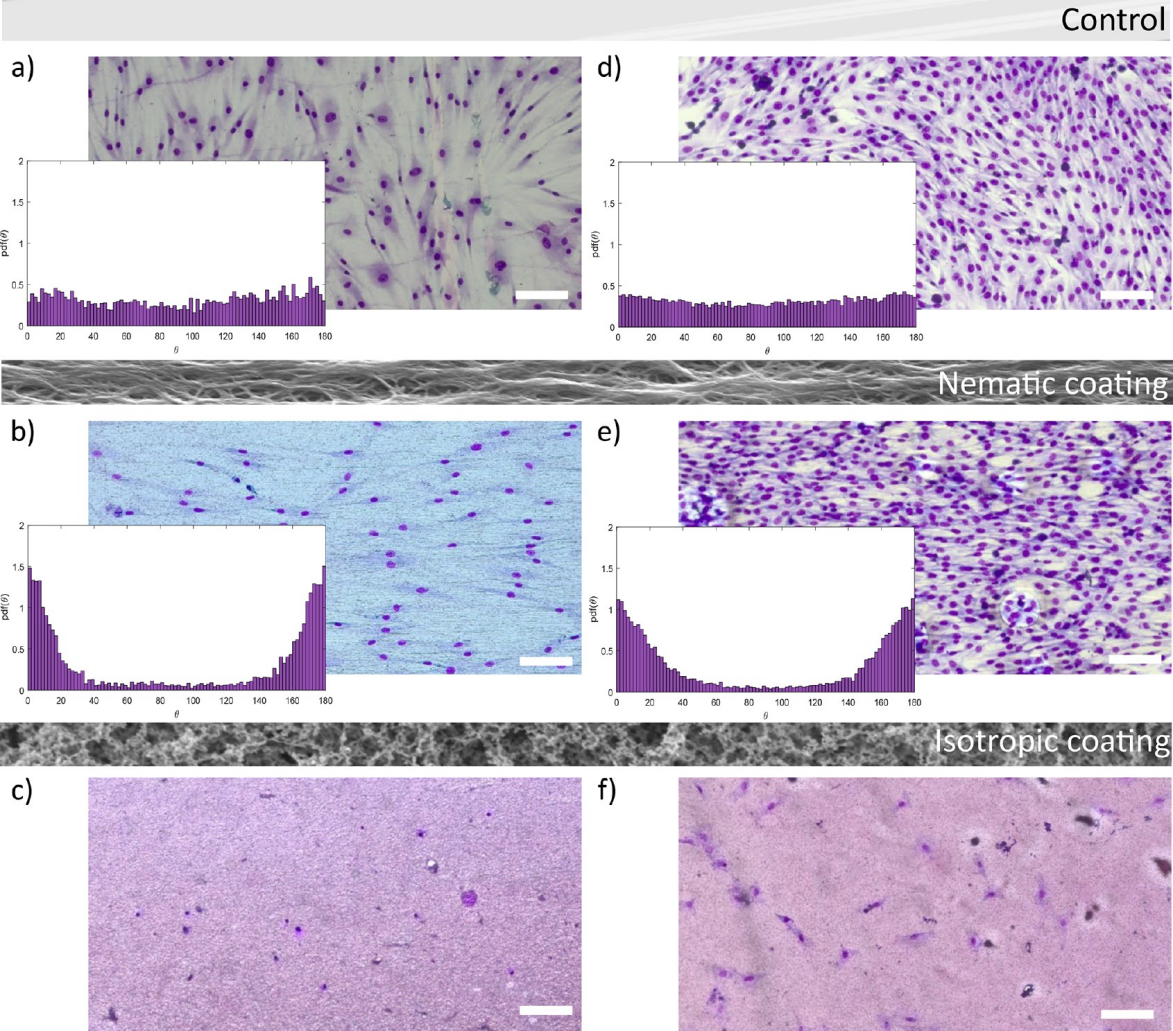
Spontaneous organization of cells on LCN coatings. Images of HDFs on (a) a Petri dish, (b) nematic coating, and (c) isotropic coating. Images of C2C12 myoblasts on (d) a Petri dish, (e) nematic coating, and (f) isotropic coating. The histograms reported the probability density function to find a cell tilted by a certain angle θ with respect to a 0° reference angle. Scale bar: 100 μm.

Then, we analyzed how the cells were organized on the different scaffolds. For each cell line, some representative images are reported in Figures 3 and S6, showing cultures at different times and with different cell densities. When present, the cell contact guidance occurred independently from the cell confluency, thus suggesting a broad applicability of these scaffolds. The cell alignment has been analyzed with a custom-made algorithm considering the major axes of the polarized (ellipsoidal) nucleus of the cell as the alignment direction.^33^ The directionality histograms reported in Figure 3 show the probability density function used to find a cell tilted with respect to a reference 0° angle, and they are calculated considering around 5000 cells for the HDFs and 15000 cells for the C2C12. The reference angle corresponds to the nematic director for the nematic coating and to the longer picture dimension for the others. For a more quantitative comparison between the samples, we also estimated the cell fraction (*f*) falling within 10° or 20° (here called f10 and f20, respectively) from the preferential alignment direction.

As expected, for both cell lines, a chaotic cell organization was observed during growth on the Petri dishes (Figure 3a,d). The alignment histogram confirmed that no direction was preferred by the cells during their growth. The situation was different on our materials. Starting from the nematic coating, a unidirectional alignment was clearly observed along the LC director, which also corresponds with the fiber orientation (Figure 3b,e). In this case, a higher degree of alignment with respect to the previously studied LCN^27^ was observed for C2C12 with f10 = 35% and f20 = 61%. For HDF, an even more uniform cell alignment was highlighted by f10 = 45% and f20 = 71%.

The same parameters, calculated for the control experiments, resulted in f10 = 11% and 11% for HDF and C2C12, respectively, and f20 = 22% and 23% for HDF and C2C12, respectively, confirming the absence of a preferred alignment.

In contrast, cells grown on the isotropic coating were able to adhere initially, but they did not proliferate and eventually died, as shown by optical microscopy images (Figure 3e,f). These results highlighted the importance of the surface morphology in the cell viability of nanostructured materials.

This set of experiments demonstrated the importance of the surface morphology derived only by the polymerization conditions and its importance for further application. Patterning the scaffold in a precise way thanks to photolithography,^38^ with polymerization in different areas at specific temperatures, we could engineer the cell adhesion only in specific parts, thus opening up to engineered cell patches with specific shapes and dimensions.

**Figure 4:**
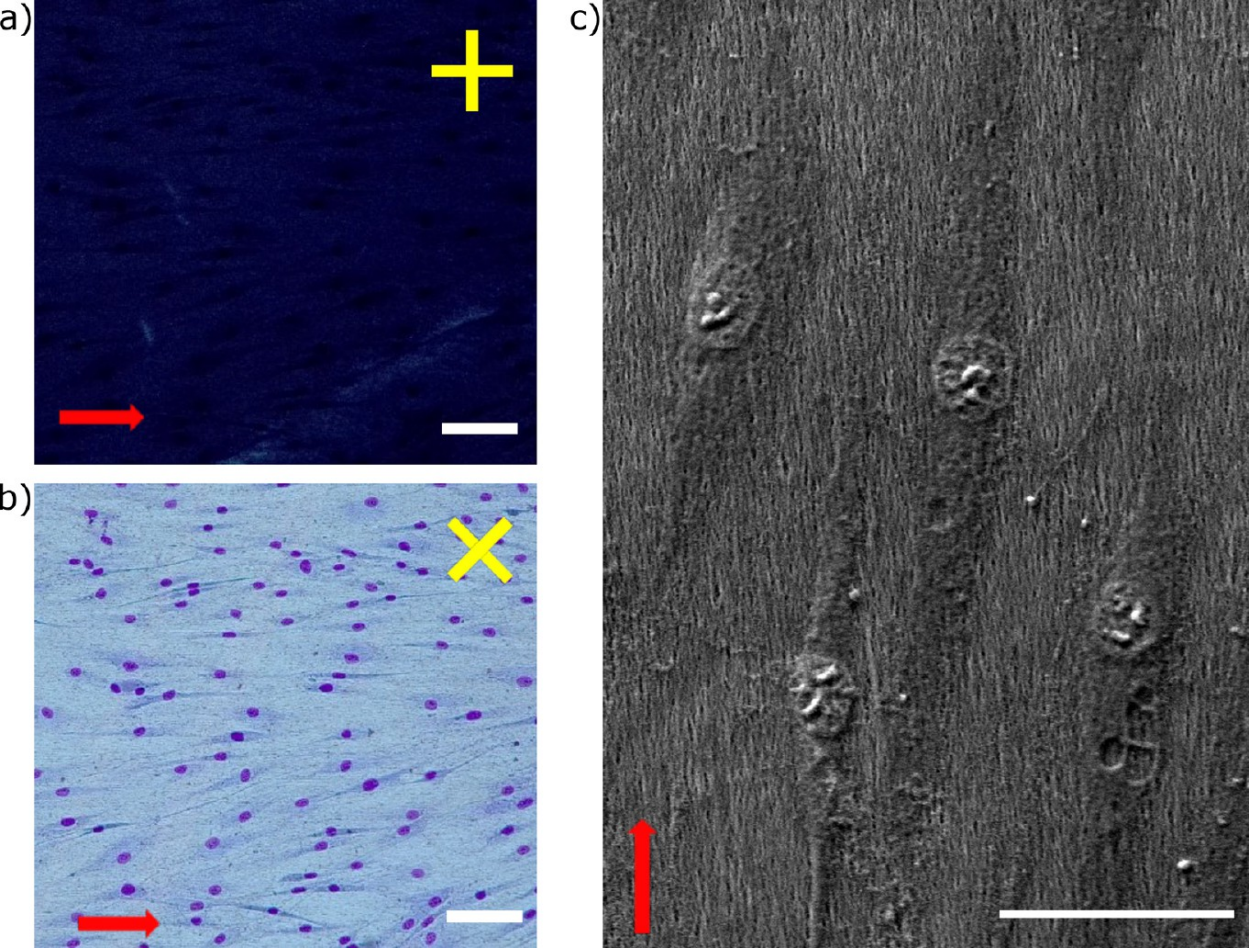
Insight on the cell alignment over LCN coating. (a and b) POM images of nematic coatings with HDFs cultured on top, as observed with the linear polarizer parallel to the LC director (a) or rotated at 45° with respect to it. Yellow bars show the linear polarizer and analyzer direction. Scale bars: 100 μm. (c) SEM image of HDFs on a nematic LCN coating. Scale bar: 50 μm. Red arrows show the nematic director.

The alignment effect of the nematic coating has been analyzed in more detail in Figure 4. Previously, some of us described the cell alignment along the LC director in flat films, but the effect was not classified as a contact guidance one. In these examples, the alignment was present only at very high confluence, thus demonstrating how the collective cell behavior pushed by the mechanical (and chemical) anisotropy of the material surface was the driving force for cell organization.^33^ However, the need of high confluence limits the possible applications for cell differentiation and for the study of other biological processes. This aspect is definitely improved by the LC coating described here.

The POM images in Figure 4a,b also highlight that at low confluences the cells align on the new LCN coatings along the nematic director, as detected by the transmittance change during rotation of the sample. The images show the same cell culture (and the same area of the sample) but with different positions of the polarizers. When one polarizer was parallel to the director of the nematic coating (Figure 4a), we observed only light extinction (dark image), while when the polarizer was rotated by 45°, a bright image was obtained (with cells also visible in this situation; Figure 4b). The transmittance variation by rotation of the sample (with maximum and minimum transmittance every 45° of rotation) is one of the features of the homogeneous planar alignment retained in the material after synthesis. Very interestingly, here cells are aligned by the scaffolds also at low confluence and at the cellular level (Figure S6), and a clear demonstration of the contact guidance due to the aligned polymeric fibers is reported in Figure 4c. The SEM image shows that both the nuclei and cytoskeleton are aligned along the LCN fibers, which behave as a good scaffold to obtain contact cell guidance for HDFs and C2C12 myoblasts.

**Figure 5:**
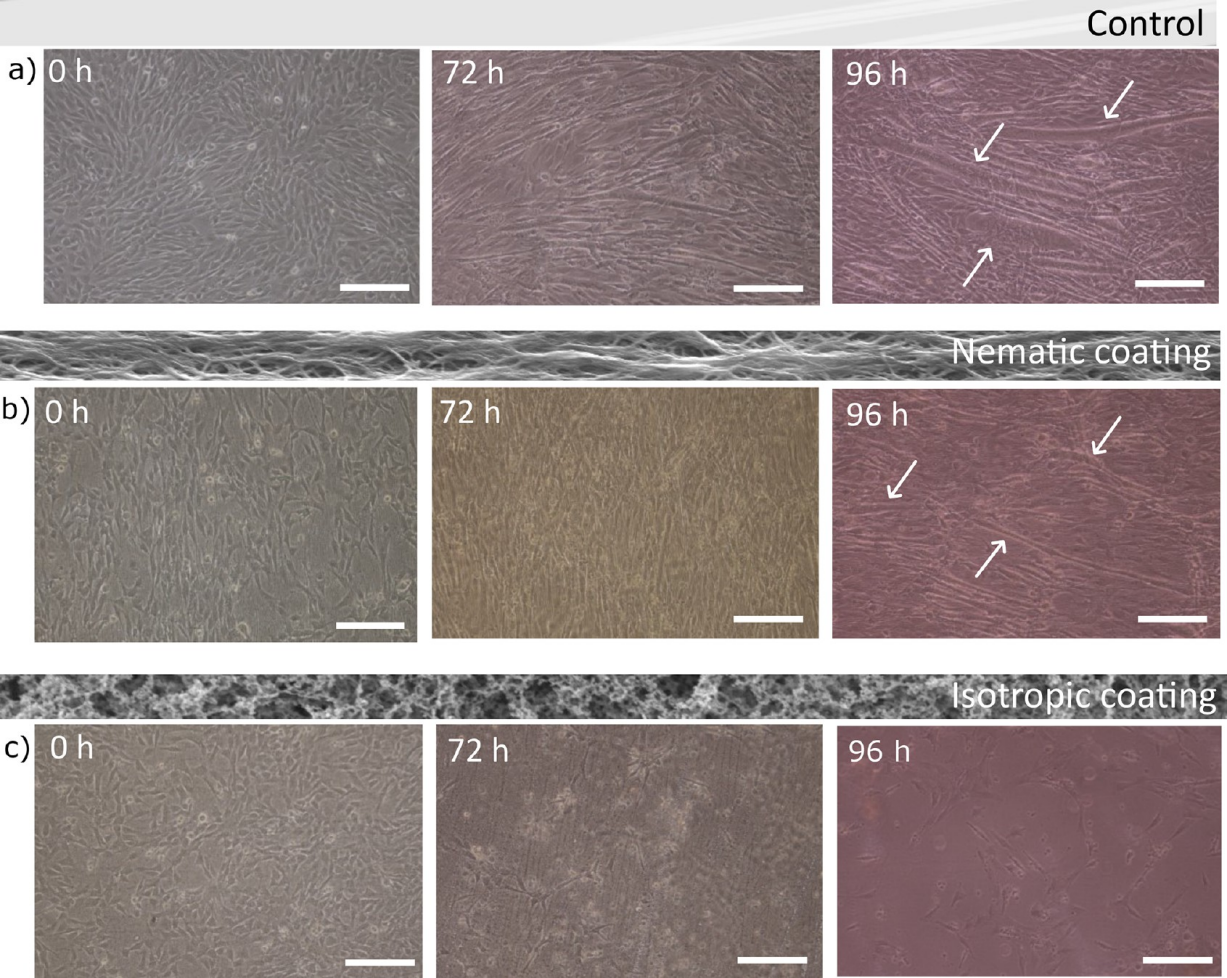
Myotube formation on LCN coatings. Representative optical images of C2C12 cells at different time points after the induction of differentiation (0, 72, and 96 h, respectively): (a) C2C12 cells on the glass coverslip used as the control; (b) C2C12 on the nematic coating; (c) C2C12 on the isotropic coating. Scale bars: 100 μm. White arrows show some formed myotubes.

**Figure 6:**
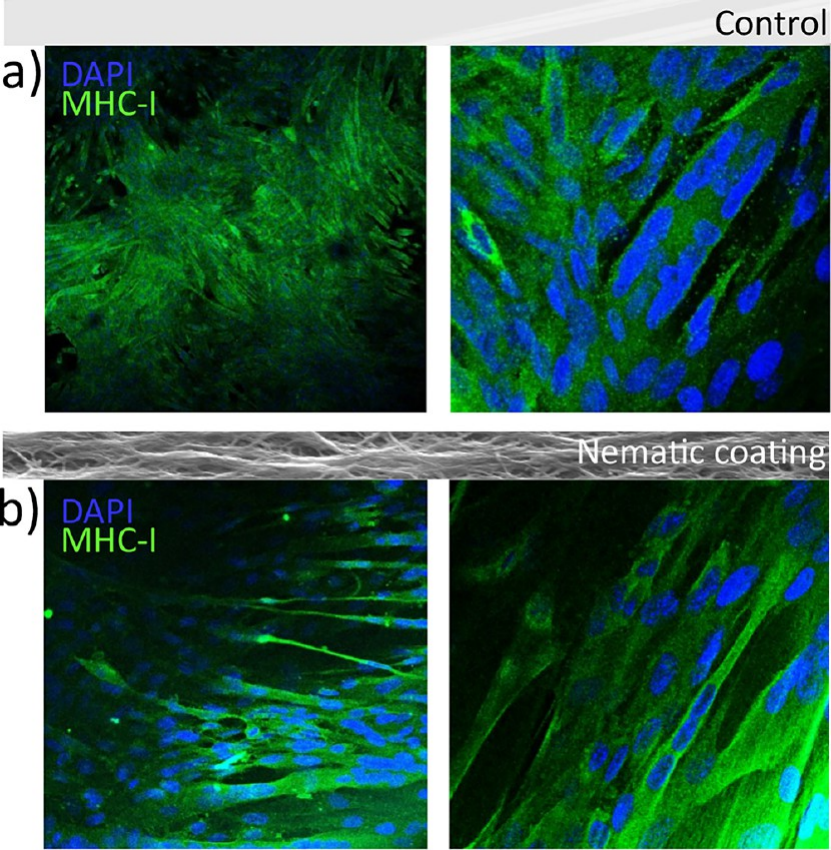
Confocal images of myotube formation on a control sample (a) and on the nematic coating (b). Green staining shows MHCs, while blue staining corresponds to the nuclei.

At the end, we tested whether the materials were able to induce myoblast differentiation into myotubes. C2C12 myoblasts have been plated again on the different LCN coatings, and after 24 h, differentiation was induced. Images of the cultures at different times are reported in Figure 5. Initially cells adhered both on nematic and isotropic coatings and on the control Petri dish. After 96 h, on the isotropic coating, the cells were not proliferating, and most of them were detached from the substrate due to cell death. On the contrary, cell fusion and differentiation in myotubes was observed on the other substrates. Microscope images highlighted that myotubes grow aligned and are mostly parallel to one another on the nematic coating, in contrast with the case of the Petri dish, where myotubes are completely randomly distributed (see also Figure S7). Using confocal microscopy (Figure 6), we verified the presence of a myosin heavy chain (MHC), as a marker of differentiation, in myotubes both on the control and on the nematic coating, with thinner myotubes having formed on the LC material. To quantify how the differentiation proceeded, an immunoblot is reported in Figure S8 for the control and two different nematic coatings. MHC expression was observed in all cases, although the control expresses higher MHC levels. Therefore, it is fundamental to improve the LC coating for further applications. Toward this objective, we prepared a different nematic coating with a polymeric network composed of 50% (w/w) RM257 and 50% (w/w) monoacrylate C6BP (Figure S8). The presence of the monoacrylate monomer led to a decreased cross-linking density and to an increased elasticity.^39,40^ In this case, a decrease in the RM257 concentration led to higher MHC expression levels, suggesting that further chemical composition development should be attempted to improve myotube formation and to guide their alignment. Some pictures of myotubes on both nematic LC coatings (containing different amounts of RM257) are shown in Figure S8, also demonstrating that the introduction of the monoacrylate facilitates the formation of larger myotubes.

## 3 Conclusion

In this paper, we demonstrated a cell guidance effect on fibroblast and myoblast cultures on anisotropic surfaces prepared starting from LCs. The materials presented different surface morphologies with nanometer features having fibrillar or rice-grain texture that can be used as a support for 2D cell culture. These different surface topographies have been prepared without the use of any lithographic techniques or printing machines but only exploiting standard microscope glasses (with coatings for planar alignment) and a UV lamp. The use of a high content of 5CB in the monomeric mixture helps to make the alignment process easier and opens up to polymerization at room temperature with possible standardization of the preparation protocol also for nonspecialist operators. The biological tests highlighted how the LCN coatings are able to control the adhesion (with the appropriate cell viability only on nematic coatings) and the organization for both HDFs and C2C12 myoblasts. Interestingly, on such substrates, cells can be well aligned also at low confluence, along the surface fibrillar structure whose direction is imprinted by the molecular LC director before polymerization. The aligning effect takes place at different cell confluences, thus demonstrating the cell contact guidance effect of the coatings. At the end, even if the differentiation process needs to be improved, myotubes can also be aligned for cell contact guidance, thus leading to the possible formation of tissues with well-structured muscular fibers. Further studies will be dedicated on modifying the chemical composition of the network to improve myotube differentiation or on combining different surface topographies in the same scaffolds to engineer cell sheets with specific shape and controlled cellular alignment.

## 4 Experimental Section

### Scaffold Preparation

Liquid-crystalline molecules were purchased from Synthon Chemicals, and Irgacure 369 was purchased from Merck. The monomer mixture was composed of 94.9% (mol/mol) 5CB, 5% (mol/mol) RM257, and 0.1% (mol/mol) Irgacure 369. The polymerization cells were made with two coated glasses, one with PVA [5% (w/w) in water] and the other with PI (Nissan Chemical Corp.), both rubbed unidirectionally with a velvet cloth. Silica beads (50 μm) were used as spacers between the glasses. The monomer mixture was infiltrated by capillarity in the isotropic phase at 95 °C on a hot plate, then cooled at room temperature to obtain the nematic phase, and irradiated with a UV LED lamp (Thorlabs M385L2-C4, 385 nm, *I* = 1.8 mW cm^–2^) for 60 min. For isotropic coating, polymerization was performed directly at 90 °C. Afterward, the cells were placed in a water bath until complete PVA dissolution and the spontaneous separation of the two glasses were reached. The material (which remained attached to the PI-coated glass) was washed with an overnight hexane bath (18 h) to remove 5CB and then dried at 50 °C.

### Scaffold Characterization

POM was performed using a Zeiss Axiolab 5 polarized microscope equipped with Axiocam 208 color and a Linkam PE120 Peltier System to control the sample temperature. A Sensofar S-Neox optical profilometer was used to measure the final coating thickness.

SEM (FEI Inspect F) was used to investigate the morphology of the samples. A Cressington sputter coater was employed to deposit a gold film (thickness of about 6 nm) on the sample surface. This gold layer makes the polymeric surface of the sample conductive, improving SEM characterization. The thickness of the gold layer was 6 nm in order to keep the morphology of the underlying sample unaltered.

AFM was used to characterize the surface roughness through topological measurements using a NanoWizard II atomic force microscope (JPK Instruments). A pyramidal AFM probe (ACTA-20-AppNano) was used to characterize the sample surfaces at intermittent mode in air on a square region with a side length of about 20 μm. To compute the 3D surface roughness, the average plane of the measured surface is subtracted from the measured surface, and *S*_a_ is computed by averaging the absolute residuals with respect to the average plane, while *S*_rms_ is computed by averaging the mean-square errors with respect to the average planes.

The AFM was also used to characterize the local Young’s moduli of the LCN scaffolds at the micrometer scale. A spherical indenter was made by binding together with an epoxy adhesive cured with UV light a tipless cantilever (TL-FM-20 by Nanosensors) and a tungsten sphere of about 10 μm diameter (357421-10G by Aldrich Chemistry). The cantilever spring constant was obtained using the thermal noise and a Sader-based method, and its value was about 5.5 N m^–1^.^41^ The cantilever’s resonance frequency was about 66.5 kHz, and its sensitivity about 33.2 nm V^–1^. The elastic modulus was measured in contact mode in an air medium over a grid of 100 points in a square with a side length of about 20 μm, and for each point, the measurement was repeated 100 times. The target applied force on the sample was 150 nN with an indentation speed of about 15 μm s^–1^ over an extended distance of 3 μm. The local Young’s modulus of the scaffolds was extracted using Hertz’s spherical punch model over the extend curves.^42^

### Cell Culture Test

The human cell line used in this study is HDFs provided by Dr. M. Calamai (LENS, Italy). The animal cell line used in this study is murine C2C12 myoblasts provided by Dr. P. Porporato (University of Turin, Italy).

The LCN coatings were air-plasma-cleaned for 10 min to smooth out the surface and subsequently sterilized by washing them three times with 70% ethanol for 15 min. The coatings were washed several times with phosphate-buffered saline (PBS), placed in 35 mm Petri dishes, and allowed to dry under a laminar flow hood. Next, approximately 60000 cells were seeded on the materials and on Petri dishes to use them as controls. Those were incubated at standard cell culture conditions (37 °C in a 5% CO_2_ humidified atmosphere) on Dulbecco’s modified Eagle’s medium supplemented with 10% fetal bovine serum. After 48 h, the LCN coatings were washed with PBS, fixed, and stained with the Hemacolor staining kit (Merck). The presence of HDFs and C2C12 adherent cells on the films was evaluated using a phase-contrast microscope. Myotube differentiation, confocal, and immunoblot analyses followed previously described methods,^35^ and they are reported in the Supporting Information.

### Statistical Analysis

The cell alignment was quantitatively estimated by statistical analysis, fully described previously,^33^ on ensembles of 5000 cells for the HDFs and 15000 cells for the C2C12 myoblasts, combining images taken at different positions on the control and nematic coating. The statistics consider that the nucleus shape is an ellipse and the tilting angle be determined with respect to a reference direction (seeing the nucleus major axis as the cell orientation direction).

## Supporting Information Available

Additional experimental details, materials, and methods, including profilometry and AFM additional analysis, POM and SEM images, and optical images of cell cultures on LCN coatings (PDF)

## Abbreviations

5CB: 4-pentyl-4-biphenylcarbonitrile
AFM: atomic force microscopy
ECM: extracellular matrix
HDF: human dermal fibroblast
LC: liquid crystal
LCN: liquid-crystalline network
MHC: myosin heavy chain
PBS: phosphate-buffered saline
POM: polarized optical microscopy
PVA: poly(vinyl alcohol)
SEM: scanning electron microscopy
