# Supplementary material for: Cellular Contact Guidance on Liquid Crystalline Networks with Anisotropic Roughness
Source: ACS Appl Mater Interfaces. 2023 Feb 15;15(11):14122–30. doi: 10.1021/acsami.2c22892 (PMC10037237; doi:10.1021/acsami.2c22892)
Supplement: Supplementary file 1 — am2c22892_si_001.pdf [file am2c22892_si_001.pdf]

Supporting Information

Cellular Contact Guidance on Liquid Crystalline Networks with anisotropic roughness

Marta Rojas-Rodríguez,[a] *Tania Fiaschi,*[b] *Michele Mannelli,*[b] *Leonardo Mortati,*[c] *Federica Celegato,*[c] *Diederik S. Wiersma,*[a,c,d] *Camilla Parmeggiani,*[a,c,e,*] *Daniele Martella.*[a,c,*]

[a]European Laboratory for Non-linear Spectroscopy, via Nello Carrara 1, 50019 Sesto Fiorentino, Italy.

[b]Department of Biomedical, Experimental, and Clinical Sciences "Mario Serio", University of Florence, viale Morgagni 50, 50143 Florence, Italy.

[c]Istituto Nazionale di Ricerca Metrologica INRiM, strada delle Cacce 91, 10135 Turin, Italy

[d]Department of Physics and Astronomy, University of Florence, via Sansone 1, 50019 Sesto Fiorentino, Italy.

[e]Department of Chemistry "Ugo Schiff", University of Florence, via della Lastruccia 3-13, 50019 Sesto Fiorentino, Italy.

camilla.parmeggiani@unifi.it
martella@lens.unifi.it

Experimental methods for myotube differentiation and analysis

Murine C2C12 myoblasts were grown in standard cell culture conditions (37 ºC in 5% CO_2 humidified atmosphere) on Dulbecco's Modified Eagle's Medium (DMEM) supplemented with 10% Fetal Bovine Serum (FBS). When the cells are in sub-confluency, in order to differentiate them, the medium was changed to DMEM supplemented with 2% Horse Serum (HOS).

Confocal analysis. C2C12 myoblasts were grown on glass coverslips as a control and on the nematic and isotropic coating until sub-confluency and then differentiated for 96 hours. The samples were washed with PBS and fixed in 3% paraformaldehyde for 20 min at 4 °C. Subsequently, the fixed samples were permeabilized with 3 washes on TBST (50 mM Tris–HCl, pH 7.4, 150 mM NaCl, 0.1% Triton X-100) and blocked with 5.5% HOS in TBST for 1 hour at room temperature. Samples were immunostained with anti-MHC primary antibody (1:100 in TBS (50 mM Tris–HCl, pH 7.4, 150 mM NaCl)) overnight at 4 °C. Then washed once with TBST, once with TBST with 0.1% BSA following the incubation with the secondary antibody conjugated with Alexa Fluor 488 (1:100 in TBS with 3% BSA). For the nuclei staining 4′,6-diamidino-2-phenylindole (DAPI) was used at a final concentration of 10 µM in TBST for 5 minutes at room temperature. Several washes with TBST were performed before mounting the samples with a glycerol mounting medium. Leica TCS SP8 confocal fluorescence microscope was used to analyze the samples.

Immunoblot analysis. Cells were lysed in 500 µl of complete radio-immunoprecipitation assay (RIPA) buffer on ice for 20 minutes. Lysates were clarified by centrifugation, and total protein contents were obtained using Bradford assay (Bio-Rad Laboratories). SDS-PAGE was used to separate total proteins for each sample (20 µg) and transfer onto PVDF membranes. These membranes were incubated in a 2% milk solution, incubated with primary antibodies, then with secondary antibodies conjugated with horseradish peroxidase.

Further information on material characterization

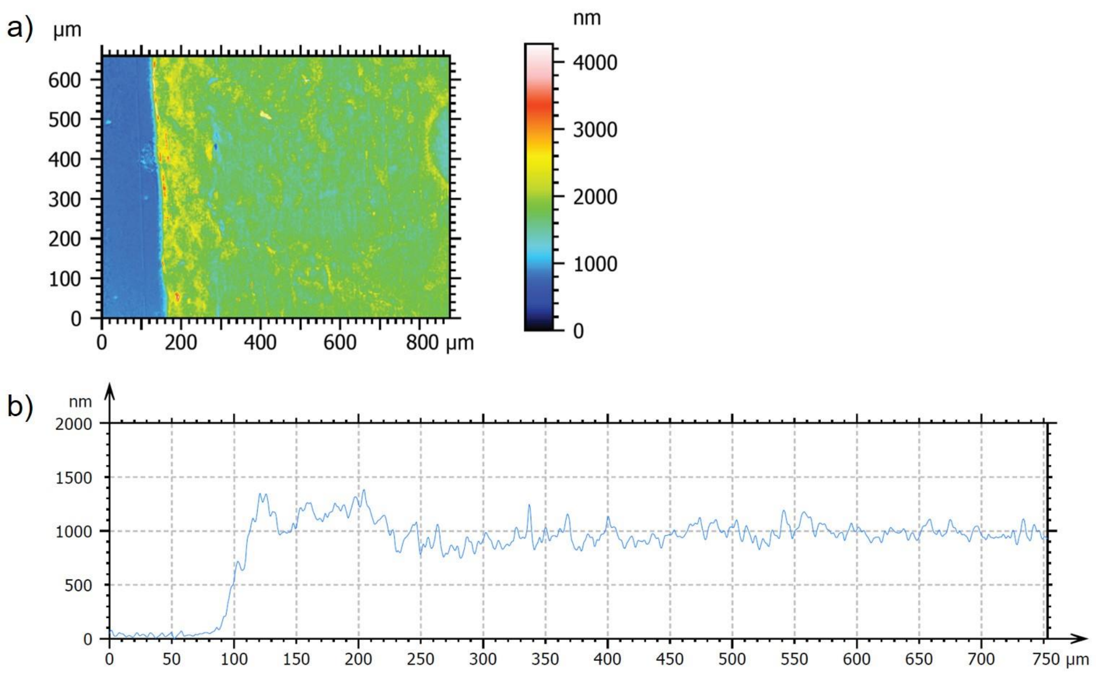

Figure S1. Profilometer analysis of a nematic coating. a) 2D height map at the border in between the glass (blue area) and the polymer coating (green area) measured by optical profilometer; b) example of height profile at the border in between the glass (corresponding to 0 nm in the y axis) and the polymer coating.

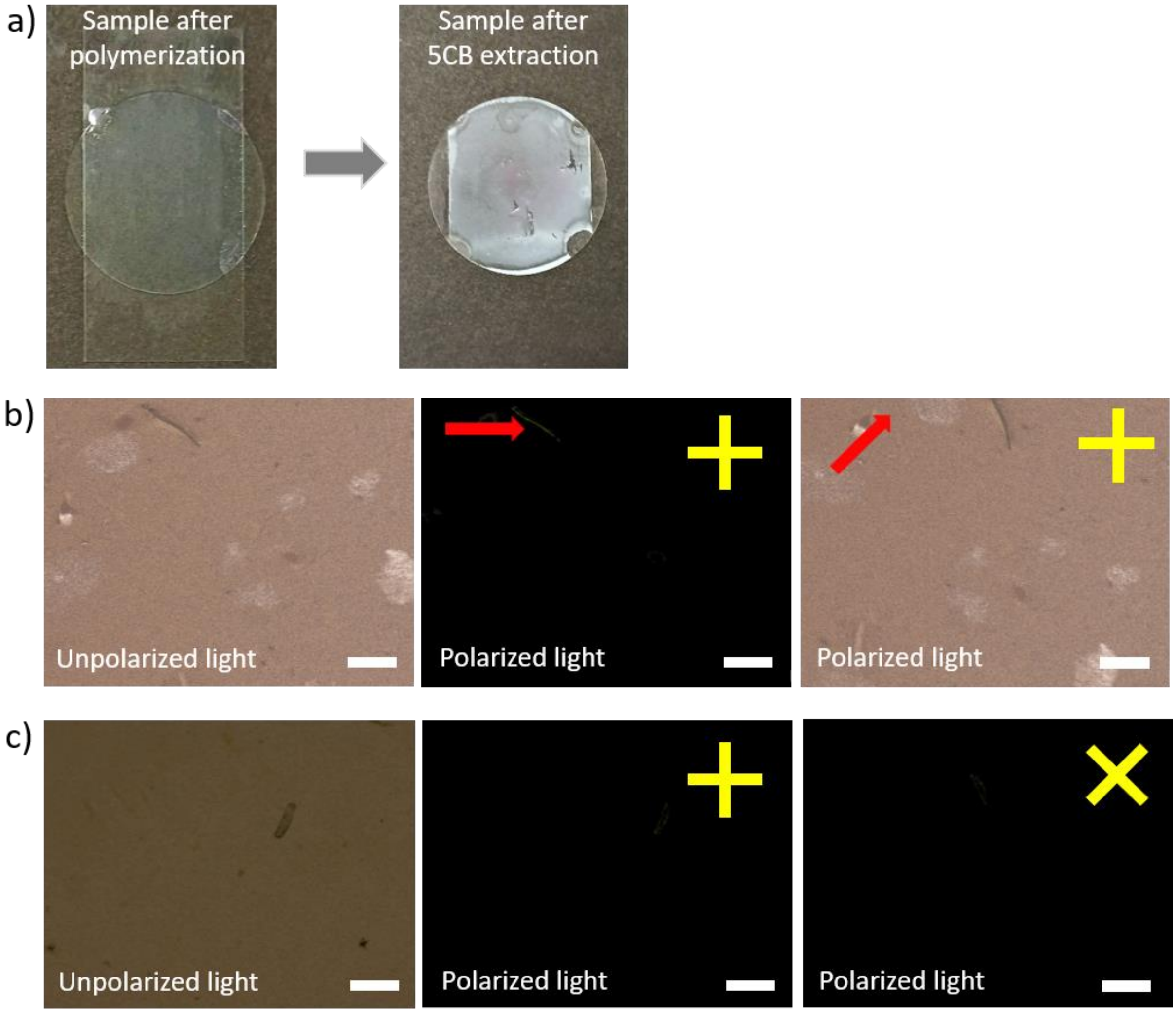

Figure S2. Details on LC alignment. a) Optical images of a nematic coating after polymerization and 5CB extraction; b) Microscope images of a nematic coating without and with polarized light; POM images under sample rotation demonstrate a transmittance change with maximum difference every 45° (as expected for a homogeneous planar alignment); c) Microscope images of an isotropic coating without and with polarized light. POM images demonstrate absence of birefringence also during rotation of the sample (as expected for isotropic materials); scale bars (in white): 100 µm, the red arrows show the nematic director and the yellow bars show the linear polarizer and analyzer directions.

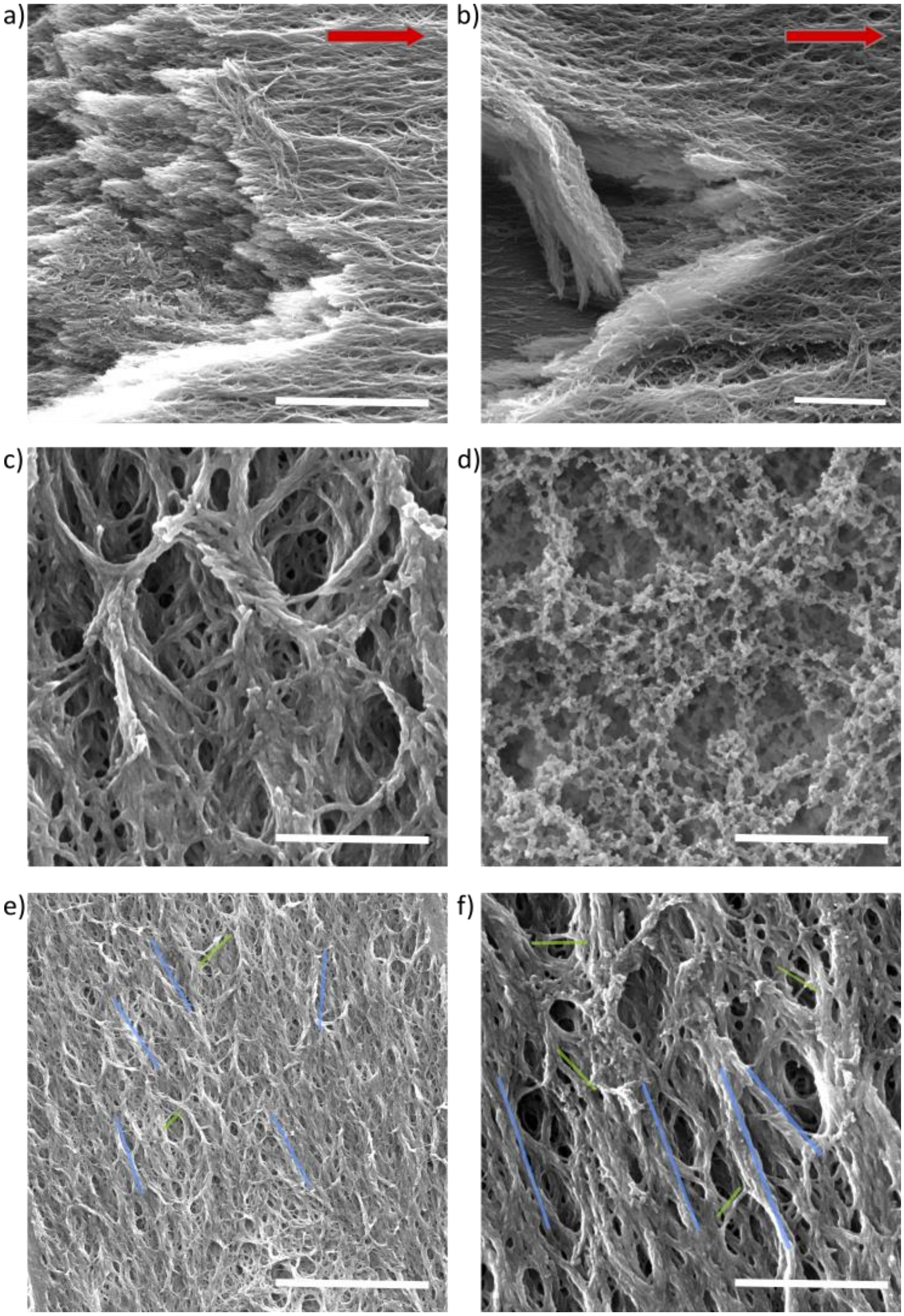

Figure S3. SEM Images for LCN coatings. a) Nematic coating observed at the border with tilted stub; scale bar: 10 μm; b) Nematic coating observed at the border with tilted stub; scale bar: 5 μm, red arrows show the nematic director; c) Nematic coating at high magnification, scale bar: 2 μm; d) Isotropic coating at high magnification, scale bar: 2 μm. e- f) SEM images of different samples of nematic coatings. Image J was used to estimate the fiber diameter report here as a medium from 3 samples. In particular, at higher magnification (f) we can observe mainly small fibers (some are highlighted in green) with diameter around 81 ± 4 nm and bigger fibers (some are highlighted in blue) with a medium diameter of 178 ± 6 nm.

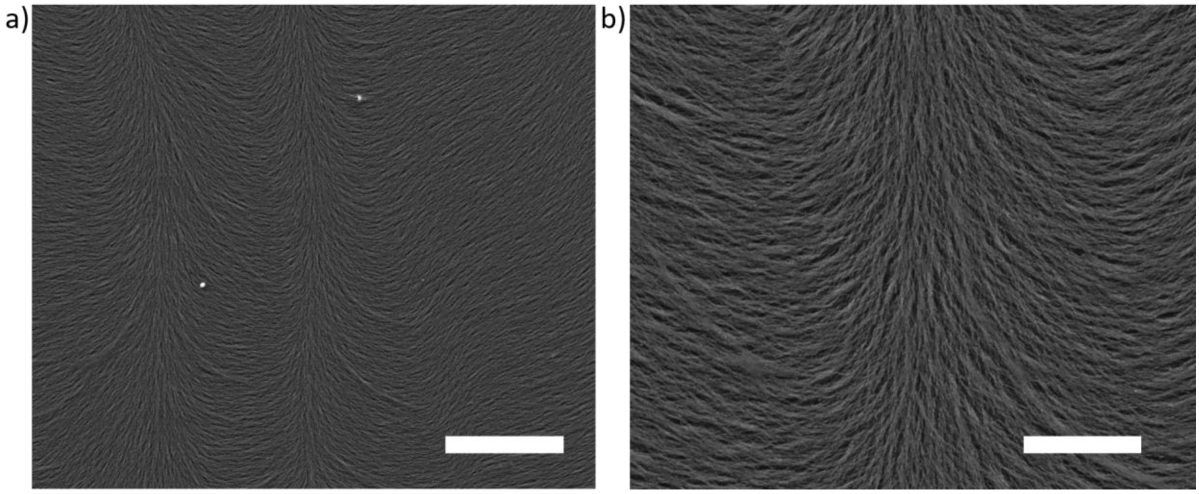

Figure S4. SEM Images for LCN coatings polymerized in a polydomain nematic sample. The nematic coating was still composed by a fibrillar structure but without an unidirectional alignment. Scale bars: 50 and 10 μm for (a) and (b) respectively.

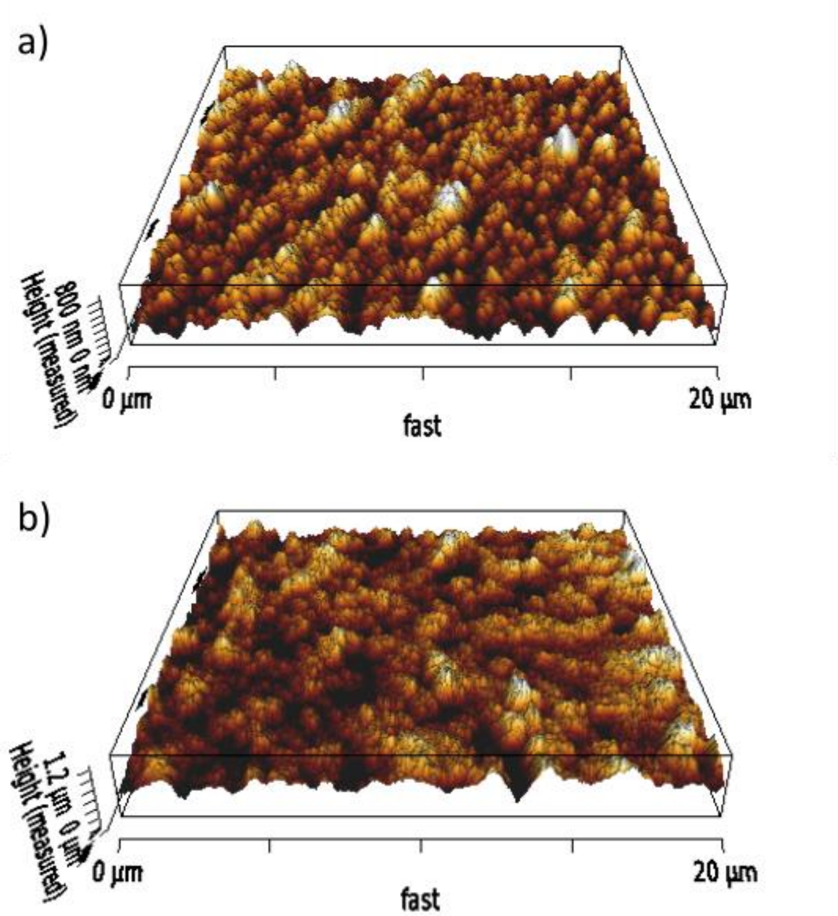

Figure S5. AFM analysis of a nematic coating (a) and an isotropic one (b).

Further information on cell culture

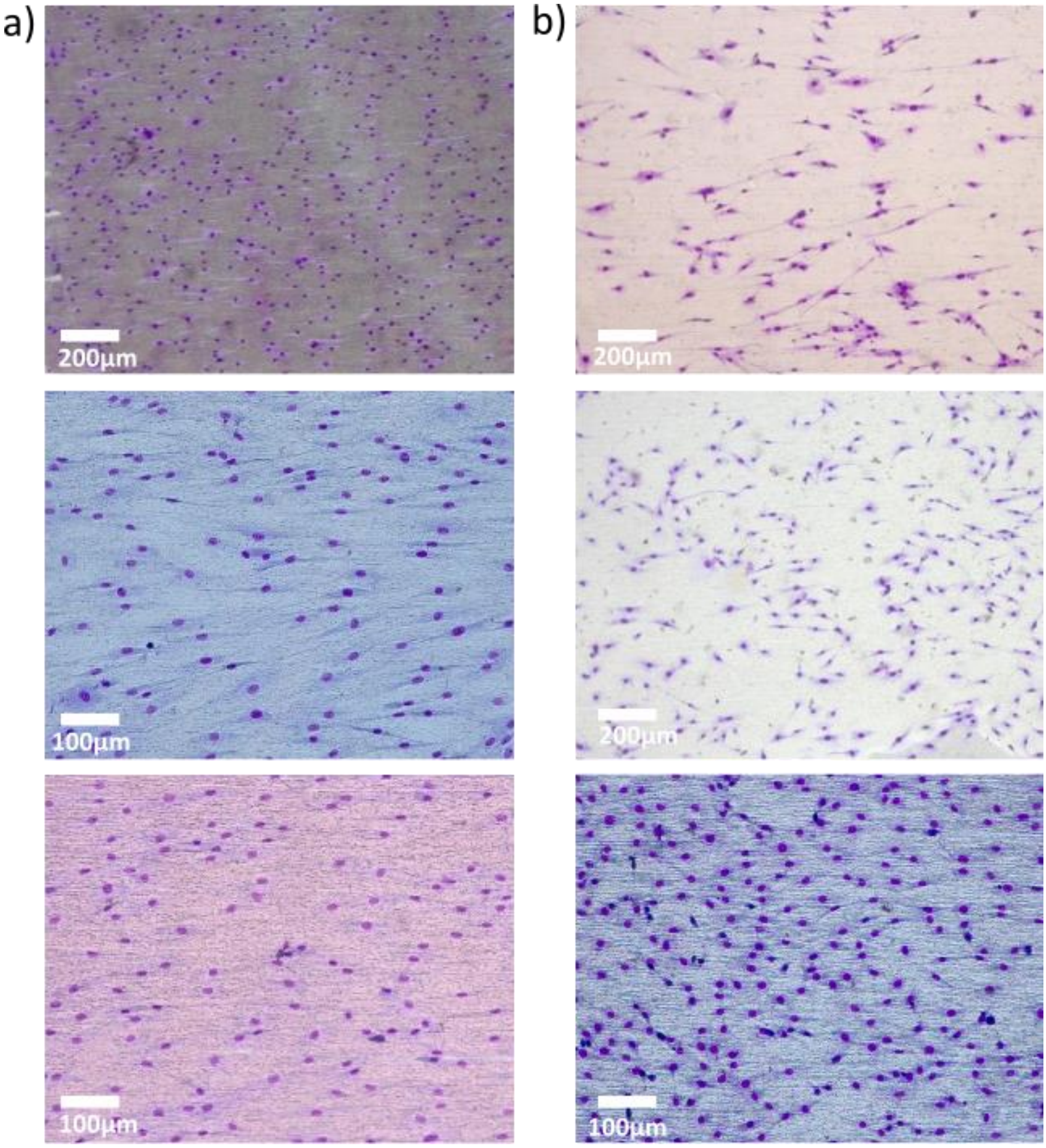

Figure S6. Other images of cells cultured on LCN coating. a) Images of HDFs on nematic coating at different magnification; b) Images of C2C12 myoblasts on nematic coating at different magnification. All images are acquired on different days (to reach different confluency levels) and tests.

Basic information on chosen cell lines. Human dermal fibroblast (HDF) is a primary cell culture from human origin. A primary cell culture is an *ex vivo* culture of cells obtained from a biopsy of the tissue of the multicellular organism (in this case the epidermis of a human). This type of culture is more representative of an *in vivo* tissue than an immortalized cell line and the cells behave in a similar way than in the living organisms. This aspect also means that, after several divisions, the cells acquire a senescent phenotype leading to a cessation of the cell division. On the other hand, C2C12 is an immortalized mouse myoblast cell line. An immortalized cell line also comes from a multicellular organism (that normally do not proliferate indefinitely) but, in this case, thanks to a mutation, the acquisition of that senescent phenotype has bypassed and the cell cycle and division keep going until some space is present on the scaffold. Those 2 different cell types were chosen because they come from different organisms (human and mouse), are widely used in research and representative of different tissues (muscular and dermal ones).

Estimation of cell density on different scaffolds. Cell density was estimated after the same number of days of culture on the different materials to compare their viability. Cells were seeded with the same density and let grow for 3 days and then stained. An area of 1 mm^2 was selected and cells were counted from 6 different images (from different experiments) on each coating and for each cell type. Then, mean and standard deviation of cell density were calculated for each scaffold (control, nematic and isotropic coating) and reported in Table S1.

Table S1. Cell density calculated for different scaffolds and each cell type.

**Scaffold**	**HDF density (cells/mm^2)**	**C2C12 density (cells/mm^2)**
Control	143±4	445±8
Nematic Coating	133±7	349±8
Isotropic coating	2±1	8±2

Further information on myotube differentiation

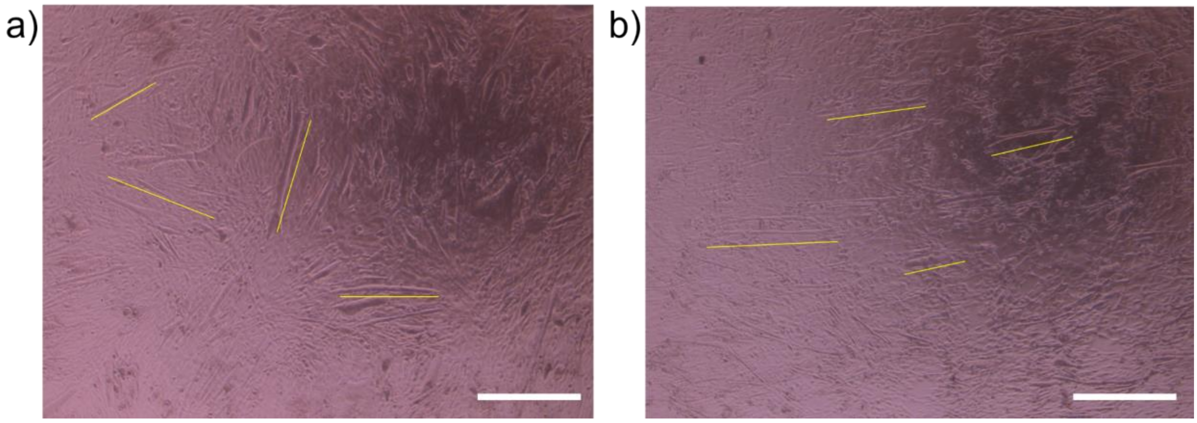

Figure S7. Optical images of myotube growth after 96 hours on the control (a) and nematic coating (b). Scale bar: 100 μm; yellow lines show selected myotube alignment directions.

a)

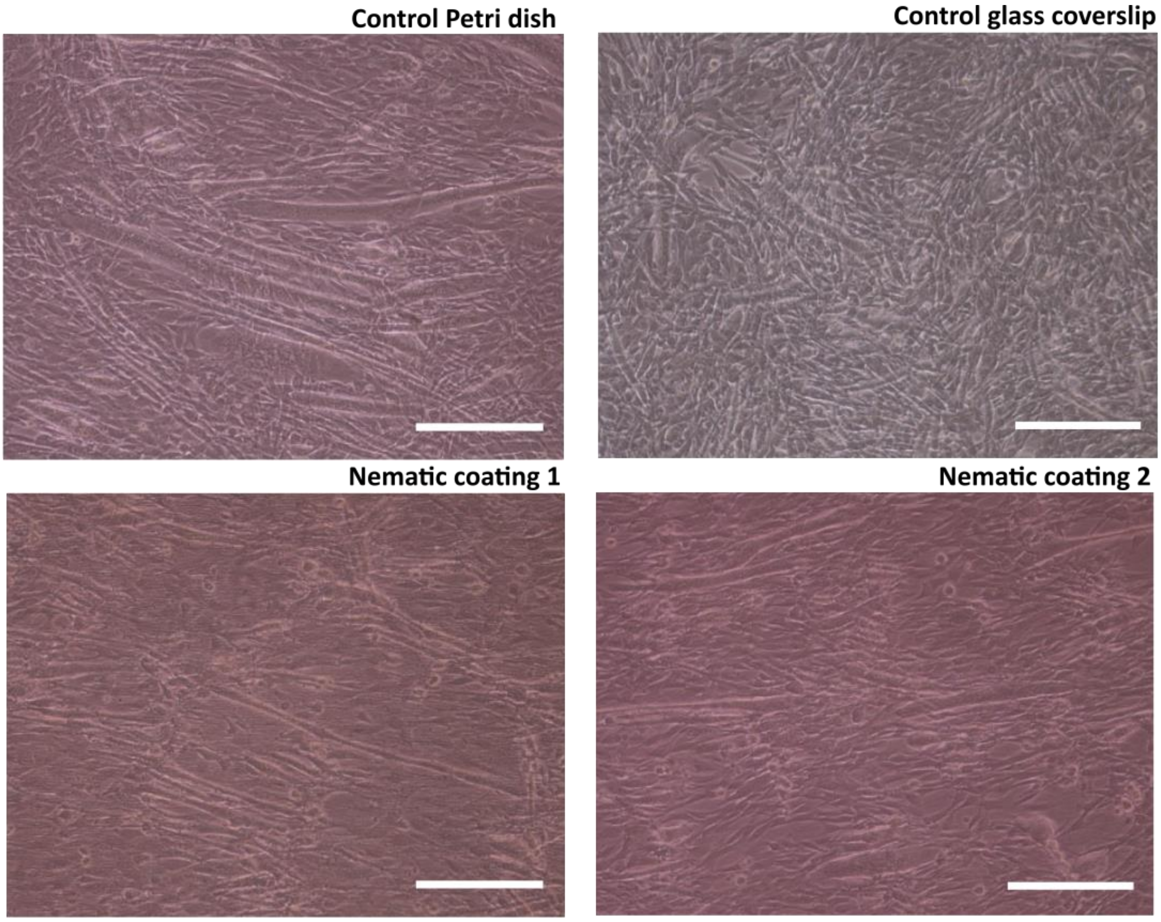

b)

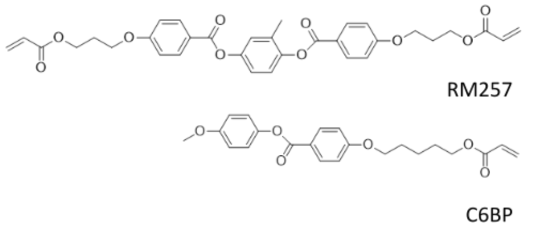

d)

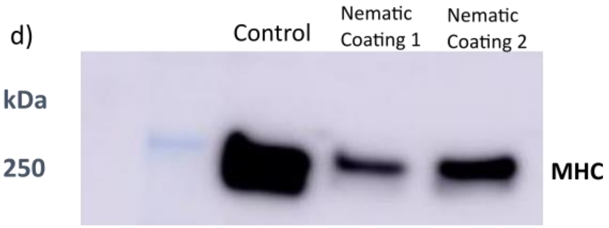

c)

	RM 257	C6BP	5CB	Irgacure 369
Nematic coating 1	5%		94.9%	0.1%
Nematic coating 2	2.5%	2.5%	94.9%	0.1%

Figure S8. Optical images and immunoblot of myotube on different nematic coating. a) Optical images of C2C12 cells 96 hours after the induction of the differentiation on different controls (Petri dish and glass) and two nematic coatings with different composition; scale bars: 100 µm; b) Molecular structure of the polymerizable mesogens used to fabricate the nematic coatings. c) Table reporting the composition of the different nematic coatings. % are expressed in mol/mol of the entire mixture. d) Immunoblot analysis of Myosin Heavy Chain (MHC) in control and different nematic coatings of the myotubes 96 hours after the differentiation.
